# Loss of Sour Taste Is the Striking Feature among Four Basic Taste Qualities in Tunisian COVID-19 Patients

**DOI:** 10.3390/jcm12020597

**Published:** 2023-01-11

**Authors:** Inchirah Karmous, Amira Sayed Khan, Imen Sahnoun, Rym Ben Othman, Houda Ben Jemaa, Faten Mahjoub, Amel Gamoudi, Leila Douik El Gharbi, Tahar Mestiri, Naim Akhtar Khan, Henda Jamoussi

**Affiliations:** 1 Research Unit on Obesity UR18ES01, Faculty of Medicine, University Tunis El Manar, 15 Rue Jebel Lakhdar, Beb Saadoun, Tunis 1007, Tunisia; 2 Centre de Recherche Inserm, U1231 INSERM/UB/Institut Agro, Team—Physiologie de la Nutrition & Toxicologie, Faculté des Sciences de la Vie, Université de Bourgogne Franche-Comté (UBFC), 21000 Dijon, France; 3 Department of Pulmonology “D”, Abderrahmen Mami Hospital, Ariana 2035, Tunisia; 4 Service «A» des Maladies de la Nutrition, Institut National «Zouhaier Kallel» de Nutrition et de Technologie Alimentaire de Tunis, 11 Rue Jebel Lakhdar, Beb Saadoun, Tunis 1007, Tunisia; 5 Service Anésthesie Réanimation, Hôpital Abderrahmen Mami, Ariana 2035, Tunisia

**Keywords:** COVID-19, objective gustatory perception, taste disorders, inflammation

## Abstract

Background: Taste disorders (TDs) have been reported to be very common in patients suffering from coronavirus disease 2019 (COVID-19), which is caused by the SARS-CoV-2 virus. In most of the hitherto conducted studies, a gustatory assessment was performed on the basis of surveys or self-reports by patients. The aim of our study was to undertake an objective assessment of four basic taste qualities by conducting tasting sessions that allowed detection thresholds in COVID-19 Tunisian patients and to study their associations with inflammation. Methods: This analytical cross-sectional study was conducted on 89 patients aged between 21 to 70 years who had been diagnosed with COVID-19. We used Burghart taste strips to assess taste perception of the four taste qualities, i.e., sour, bitter, sweet, and salty. Serum levels of interleukin-1β (IL-1β), interleukin-6 (IL-6), tumor necrosis factor alpha (TNF-α), and C-reactive protein (CRP) were measured. Results: Taste disorders were reported by 40.4% of the patients, while objective assessments revealed that 63.8% of participants were suffering from hypogeusia and/or ageusia. Sour taste was the most altered (70.8%) gustatory quality. Patients with severe COVID-19 had significantly lower sour and bitter taste scores when compared to patients with minor/moderate forms. There was no significant association between serum inflammatory markers and taste disorders. However, the relationship between bitter and sweet taste qualities and IL-1β levels was significant (*p* = 0.018 and *p* = 0.041). Conclusions: Our results demonstrate the interest in the objective assessment of taste dysfunctions in COVID-19 patients.

## 1. Introduction

Since the declaration by the World Health Organization (WHO) of the global pandemic in March 2020, coronavirus disease 2019 (COVID-19) has spread in several episodes through almost every country in the world. The novel coronavirus, SARS-CoV-2, like other RNA viruses, is susceptible to genetic evolution as it adapts to its new human hosts with the development of mutations over time, leading to the emergence of multiple variants that may have different characteristics compared to their ancestral strains. Therefore, the clinical manifestation of a SARS-CoV-2 infection is highly variable. The most common symptoms of COVID-19 are fever, fatigue, dry cough that is often accompanied by sputum production, headache, anorexia, and sore throat [1,2]. Although the symptoms of COVID-19 are predominantly respiratory in nature, symptoms and complications have increasingly been described in the central and peripheral nervous system, including anosmia and ageusia [3,4]. An altered sense of taste, in its various manifestations (ageusia, dysgeusia, or hypogeusia), generally occurs within 3–5 days of the clinical onset of the disease. A perturbation in taste perception is related to the clinical form of SARS-CoV-2 infection. Early reports in 2020 showed that taste perception was more frequently altered in mild forms of coronavirus disease compared with that in severe forms [4,5]. Data from systematic reviews and meta-analyses showed that the prevalence of an altered sense of taste varied from study to study, ranging from 1% to 93% [1]. The high variability may depend on gender, age, the severity of the disease, country of origin, or ethnicity, as well as on differences in examination methods [6].

In a Tunisian retrospective study carried out between March and May 2020 among 1030 subjects with COVID-19, taste disorders were reported in phone call interviews in 27.4% of the cases [7]. Another Tunisian study conducted in the same period found that 36.8% reported taste impairment [8]. To avoid the risk of viral contamination, taste disorders were recorded in most cases by using subjective methods, such as surveys or self-reports. However, a few studies [9,10,11,12,13,14,15] have focused on objective assessments, and not much is known about the characteristics of taste loss.

The inflammatory response generated by SARS-CoV-2, which is mediated by the virus via toll-like or other receptors, stimulates pro-inflammatory cytokines that trigger the inflammatory process and result in apoptotic cell death, causing an abnormal renewal of the taste buds and, therefore, taste disorders [16,17]. Some authors have suggested the role of inflammation in taste dysfunction by reporting an increase in the level of interleukin-6 [18,19].

By keeping the above-mentioned arguments in consideration, we designed the present study to specify which of the four tastes (sweet, salty, bitter, and sour) was altered in relation to inflammatory status in Tunisian COVID-19 patients.

## 2. Materials and Methods

### 2.1. Study Population

This multicentric cross-sectional study was carried out on 89 SARS-CoV-2-positive patients in the departments of pulmonology “D” at Abderrahmen Mami Hospital and nutritional diseases “A” at the National Institute of Nutrition and Food Technology (INNTA) in Tunis between November 2020 and July 2021.

To be eligible for the study, patients had to meet the following inclusion criteria: They were adults over 18 years of age, were diagnosed with COVID-19 by an RT-PCR test, and reported clinical symptoms of COVID-19 infection.

We did not include the patients with a history of taste disturbances before COVID-19 infection, decreased consciousness, confusional state, and cognitive impairment that prevented reporting symptoms in our study. Patients requiring hospitalization in intensive care units or resuscitation, pregnant and lactating women, and smokers were not included.

This study was conducted in accordance with the Declaration of Helsinki (1989) of the World Medical Association, and it was approved by the ethical committee of the Tunisian National Institute of Nutrition and Food Technology. Free and informed consent was obtained from each patient before the start of the study. All participants signed an informed consent form.

### 2.2. Study Design

At the time of diagnosis of COVID-19, demographic information, including age, gender, medical history, weight, height, and body mass index (BMI), was obtained from the study participants. Normal weight, overweight, and obesity were defined as BMI ranges of 18.5–24.9, 25–29.9, and ≥30 kg/m^2^, respectively [20].

Interviews were performed to ask patients about the symptoms of COVID-19, including smell and taste disorders. Clinical forms of the COVID-19 disease were defined according to the Tunisian National Authority for Evaluation and Accreditation in Health (INEAS) criteria [21] as follows: minor form (no pneumonia, mild dry cough, dizziness, headache, muscle pain, smell and/or taste alteration, no dyspnea), moderate form (pneumonia without signs of severity (cough, mild dyspnea, RR < 30 cpm, SpO_2_ ≥ 94%)), and severe form (dyspnea, RR ≥ 30 cpm, and/or SpO_2_ < 94% in ambient air).

### 2.3. Sample Collection and Determination of Inflammatory Markers

On the day of COVID-19 diagnosis, blood samples were collected and centrifuged (1000× *g*, 15 min, 4 °C), and serum was extracted and immediately stored at −20 °C until the analysis. We measured the circulating concentrations of inflammatory cytokines, i.e., interleukin-1β (IL-1β), interleukin-6 (IL-6), and tumor necrosis factor alpha (TNF-α), with ELISA (Raybio tech Inc., Norcross, GA, USA). C-reactive protein (CRP) was analyzed with routine standard techniques by using a Synchron CX7 automated Clinical System (Beckman Coulter Inc., Brea, CA, USA).

### 2.4. Tasting Sessions

The assessment of objective gustatory dysfunction (GD) was performed with a tasting strip test (Burghart Messtechnik GmbH, Wedel, Germany). This validated test contained 18 spoon-shaped strips impregnated with four different concentrations of sweet, sour, salty, and bitteras well as two blanks [22,23]. Each strip had to be placed in the middle of the tongue. To check the gustatory sensitivity of different areas of the tongue, the strips were brought into contact with the respective areas until patients could give a response. Subjects had to choose one of the following answers: “no taste”, “sweet”, “sour”, “salty”, or “bitter”. One point was granted for each correct answer. The maximum number of points, therefore, was 4 points per taste quality or 16 points for the whole test. In addition to the impregnated strips, two tasteless strips were integrated into the examination at the beginning and the end of the test (no points). After each taste strip, the mouth was thoroughly rinsed with water. A maximum of 16 points could be achieved. A score below the nine-point threshold was referred to as hypogeusia, and the absence of sensation in any of the taste qualities was referred to as ageusia. Furthermore, hypogeusia (1 or false identification) or ageusia (no sensation) could only measure certain taste qualities.

### 2.5. Statistical Analysis

Data were analyzed with the IBM SPSS statistics for windows, version 21.0 software, Armonk, NY, USA, IBM Corp. The results were presented as the mean ± standard deviation (mean ± SD) for continuous variables and percentages (%) for categorical variables. We used an independent *t*-test to compare the means. The chi-square test was used for the comparison of percentages. In this study, *p* < 0.05 was considered statistically significant.

## 3. Results

Eighty-nine participants were eligible to participate in the study based on the inclusion criteria. The mean age of the study participants was 49.69 ± 12.44 years. A total of 53 participants were women, accounting for 59.6%. The average BMI of the patients was 30.13 ± 7.26 kg/m^2^. The general characteristics of the population are presented in Table 1.

Two-thirds of the study population (67%) had a severe form of the disease according to the INEAS diagnostic criteria.

Subjective taste disturbances were reported by 36 patients (40.4%). The objective assessment of the taste perception with the strips revealed that 63.8% of the patients had ageusia or hypogeusia. The difference between subjective and objective assessments of GD was statistically significant (*p* = 0.04).

The taste assessment showed a discrepancy between the subjective and objective taste discrimination. Indeed, among 53 patients who did not report taste alterations, 35 patients actually had ageusia and/or hypogeusia. On the other hand, 16 of the 36 patients who had reported an alteration in taste had normal taste sensation as assessed with our strip method.

According to the total score, men were more affected by taste dysfunction than women were (5.94 ± 2.94 vs. 8.11 ± 3.11; *p* < 0.001). Ageusia and hypogeusia patients were older (53.38 ± 10.07 years) than normogeusia patients (43.73 ± 13.69 years; *p* < 0.001).

The BMI of the ageusia and hypogeusia patients was higher than the BMI of the normogeusia patients (31.85 ± 7.80 kg/m^2^ vs. 27.07 ± 5.15 kg/m^2^; *p*< 0.002). The mean score was significantly lower in subjects with obesity compared to normal-weight subjects (6.14 ± 3.08 vs. 8.94 ± 2.52, *p* = 0.003). Subjects with obesity were more affected by taste dysfunction than subjects with normal weight (Table 2). The mean scores of the sweet (2.23 ± 1.32 vs. 3.15 ± 1.01; *p* = 0.040) and bitter tastes of obese patients (1.41 ± 1.30 vs. 2.35 ± 1.39; *p* < 0.01) were lower than those of subjects with normal weight. The serum concentrations of inflammatory markers (IL-1β, TNF-α, and CRP) were higher in subjects with ageusia/hypogeusia than in subjects with normogeusia, but without any statistical significance.

**Figure 1:**
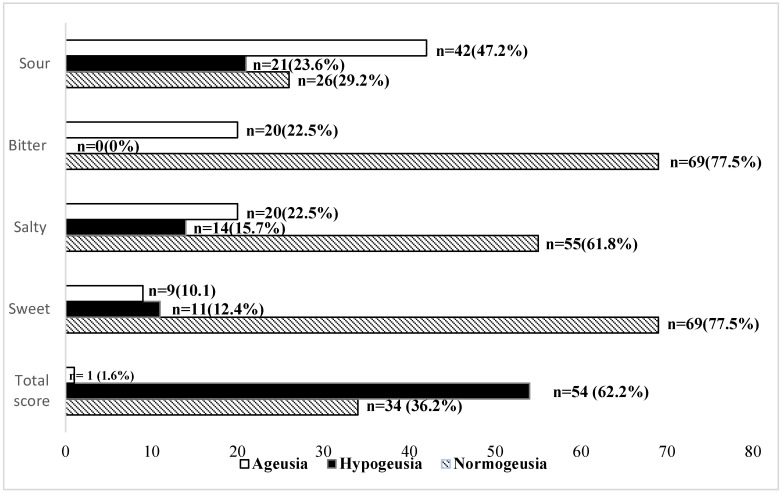
Distribution of the population by taste test scores.

The evaluation of the objective perception of the four flavors revealed that the sour taste was the most altered. Indeed, ageusia and/or hypogeusia were observed in 70.8% of the cases for sour taste, 38.2% of the cases for salty taste, and 22.5% of the cases for sweet and bitter tastes (Figure 1).

Sour and bitter tastes were significantly more altered in patients who had a severe form of COVID-19 (Table 3).

## 4. Discussion

This is the first Tunisian study to focus on the objective assessment of taste disorders in patients with COVID-19. Taste strips impregnated with four tastes (sour, bitter, sweet, and salt) were used for this evaluation. Our results showed that, when assessed objectively, taste disorders were more frequent (63.8%) than when reported by the patients (40.4%). The self-report of dysgeusia was not very reliable, since the taste test revealed that among 53 patients who did not report taste alterations, 36 patients actually had taste dysfunction.

The Burghart strip test that was used in our study is a validated method that objectively evaluates gustatory dysfunction. This method was used by some authors, while others preferred taste solutions instead of strips [24].

A recent literature review that was published in March 2022 and that focused on taste and smell disorders in patients with COVID-19 showed that the prevalence of taste dysfunctions varied from country to country [25]. The highest frequency was observed in Spain (83.9%) [26]. The data on the prevalence of subjective taste disorders varied between 33.9% and 88.8% [10].

Some authors observed significant differences between subjective and objective taste perception [10], while others did not [11,27,28]. Burghart’s taste strips provide a good tool for an initial clinical assessment of taste loss, especially with regard to ageusia; however, the fine nuances of hypogeusia cannot be detected easily and could, thus, also be part of the reason for why the subjective and objective data differed in our cohort [10].

This present study allowed us to identify the most altered taste qualities, which were “sour” followed by “salty” in the second position. Our observations corroborate the findings of Singer-Cornelius et al., who also reported the same results in patients with COVID-19 [10]. Both tastes are transmitted via ion channels, while bitter and sweet qualities are taste qualities that are transduced by a G-protein-coupled receptor [29].

To explain the pathophysiological mechanisms of taste alteration, several hypotheses have been proposed; angiotensin-converting enzyme 2 (ACE2) was identified as a cellular receptor for SARS-CoV-2 [24]. ACE2 receptors are expressed in the tongue epithelium [17,30]. The role of ACE2 in modulating taste perception has been highlighted in many studies examining the chemosensitive side effects of ACE2 inhibitors and angiotensin-II blockers [31]. El Kady et al. [32] revealed that taste disturbances in COVID-19 patients could be explained by a viral infection of the olfactory cranial nerves and/or nasal obstruction, with which taste and smell disturbances are closely associated, and they are often reported together.

Zinc deficiency is another mechanism of dysgeusia proposed by some authors. This hypothesis was developed because zinc is an important mineral that is essential for maintaining the sense of taste. Jothimani et al. [33] reported that the zinc levels in SARS-CoV-2-infected patients were significantly lower than those in healthy controls, thus explaining the alterations in taste in the latter. Not only systemic, but also local tongue inflammation may contribute to ageusia. The deficiency of zinc, which is related to high inflammation, may also be responsible for taste dysfunctions, as suggested by Rathee and Jain [34], though we did not determine the zinc concentrations in COVID-19 patients [35].

It Is also interesting to know that SARS-CoV-2 can cause taste disorders by interacting with sialic acid receptors, which play a central role in taste perception. Indeed, Milanetti et al. reported that SARS-CoV-2 could interact with the sialic acid receptor, thus inducing an increased taste threshold for tastants [36].

Our results demonstrate that disease severity, gender, age, and BMI significantly influence taste perception. Thus, hypogeusia and ageusia for sour and bitter tastes were observed in almost 80% of the cases with a severe form of the disease. Our results are in close agreement with the report of dos Santos et al. [37], who showed that taste alteration was more frequently found in individuals with a severe form than in those with a minor or mild form of SARS-CoV-2 infection. On the other hand, unlike in the Brazilian study, in their study that was conducted in May 2020, Lee et al. [38], found that taste alteration was rather common in patients who had developed a mild to moderate form of COVID-19 infection. These symptoms probably have a pathological basis related to neurotropic infection in the gustatory or olfactory systems [11].

As far as gender is concerned, Cazolla et al. [18] and Asadi et al. [39] reported that men exhibited lower taste test scores than women did. On the other hand, contradictory results were reported by two studies showing that ageusia was higher in women than in men [40,41]. Taste preference, taste recognition thresholds, and reactivity to taste stimuli are also known to be influenced by gender differences. It is believed that this happens at the level of the taste buds. Behavioral and neurophysiological evidence pointed out that such differences occur throughout the taste system and that sex steroid hormones may modulate taste processing in the brain, as sex hormone receptors appear to be prominent in multiple nuclei associated with central taste pathways. In particular, estrogen levels can affect taste-induced activity in the periphery and brainstem, particularly in the limbic pathway [42]. Brion et al. [43] stressed that chemosensory dysfunctions—both olfactory and taste functions—are altered in many neurological and psychiatric states.

Age also plays a key role in taste alteration in patients with COVID-19. Taste alteration increases with age, as reported by Hannum et al. [27]. Nevertheless, in their study published in May 2020, the results of Lee et al. [38] demonstrated that taste alteration was more frequent in young individuals. In other studies that were carried out in Turkey and Italy, no significant association between age and altered taste was observed [40,44]. Loss of smell is more common in older individuals, as we observed in our study [45].

In addition to the severity of the infection, gender, and age, obesity is also considered a factor that can alter the taste perception of COVID-19 patients [17]. However, a Saoudian study did not find an effect of BMI on taste alterations [6]. The association between the alteration of bitter and sweet tastes and BMI could be partly explained by the alteration of these two tastes in obese subjects reported in the literature. One of the mechanisms of this alteration is the mutation of the genes of the receptors for these tastes. Obesity has been shown to be associated with low oro-naso-sensory cues. These alterations in subjects with obesity are due to obesity-induced altered expression of olfacto-taste receptors. Furthermore, obesity can further aggravate SARS-CoV-2 infection, as this pathology is associated with high levels of inflammation/immunosuppression and reduced protection against viral infections [17].

Generally, inflammatory markers decrease viral replication by recruiting other immune cells to fight infections. On the other hand, uncontrolled production of cytokines can cause real damage. Indeed, the significant elevation of IL-6 can inhibit the function of smell through the activation of apoptotic circuits by using TNF-α. In addition, a high level of IL-6, which represents the key cytokine in the event of viral infection, activates the intravascular coagulation cascade, which is also associated with more severe clinical manifestations of SARS-CoV-2 infection [18].

The alteration of bitter and sweet tastes was significantly associated with an increase in the level of IL-1β levels in our study. This association may be mediated by vitamin D [46]. In the literature, only one study investigated the relationship between taste disorders and inflammation in COVID-19 patients. Indeed, Cazzolla et al. [18] showed a link between taste alteration and cytokine storms. Indeed, the average level of IL-6 was significantly higher in patients with taste disorders (*p* = 0.047) [18]. IL-6 concentrations were elevated in patients with severe taste disturbance and positively correlated with taste loss scores in several studies [46]. Indeed, pro-inflammatory cytokines may have directly affected taste bud functions, resulting in taste disorders.

Strengths and limitations of the study: This is the first Tunisian study to focus on the objective evaluation of taste disorders in COVID-19 patients, and it involved a fairly large sample (89 patients). In addition, we could not realize taste detection sessions after the recovery of the patients, who were not reachable.

## 5. Conclusions

This study, the first in Tunisia, emphasized the interest in the objective evaluation of taste disorders in COVID-19 patients. Although inflammation is associated with severe forms of the disease, it partially explains the alteration in taste qualities. The loss of sour taste was the predominantly change in taste in Tunisian COVID-19 patients.

## Figures and Tables

**Table 1:** Detailed representation of demographic data.

	*n*	%
Gender		
Male	36	40.4
Female	53	59.6
Age		
20–35 years	11	12.4
35–50 years	29	32.6
50–65 years	36	40.4
<65 years	13	14.6
BMI * class		
Normal weight	19	21.3
Overweight	28	31.5
Obesity	42	47.2
Antecedents		
Type 2 Diabetes	22	18
Allergic rhinitis	2	2.4
Asthma	3	3.7
Sleep apnea syndrome	4	4.9
COVID-19 symptoms		
Fever	47	52.8
Headache	34	38.2
Cough	38	42.7
Asthenia	48	53.9
Anosmia	34	38.2

* BMI: Body Mass Index.

**Table 2:** Clinical and biological factors influencing taste perception.

	Ageusia/Hypogeusia (*n* = 55), (%)	Normogeusia (*n* = 34), (%)	*p* Value
Gender Male (*n* = 36) Female (*n* = 53)	28 (50.9) 27 (49.09)	8 (23.52) 26 (76.47)	ns
Age (years) 20–35 (*n* = 11) 35–50 (*n* = 29) 50–65 (*n* = 36) <65 years (*n* = 13)	1 (9%) 19 (65%) 25 (69%) 10 (76%)	10 (91%) 10 (35%) 11 (31%) 3 (24%)	0.01
BMI class Normal weight (*n* = 19) Overweight (*n* = 28) Obesity (*n* = 42)	6 (11) 16 (29) 33 (60)	13 (38) 12 (35) 9 (27)	0.003 ns 0.002
COVID-19 form Minor/Moderate (*n* = 30) Severe (*n* = 59)	15 (27.27) 40 (72.72)	15 (44.11) 19 (55.88)	ns
Markers of Inflammation IL-1B (pg/mL) IL-6 (pg/mL) TNF (pg/mL) CRP (mg/L)	Mean ± SD 8.05 ± 8.39 27.16 ± 65.2 1.39 ± 0.49 88.36 ± 73.28	Mean ± SD 7.22 ± 2.00 28.44 ± 43.18 1.33 ± 0.42 78.23 ± 71.54	ns ns ns ns

The serum concentrations of the inflammatory markers (IL-1β, TNF-α, and CRP) were higher in ageusia/hypogeusia patients compared to those in normogeusia participants, but without any statistical significance; ns: no significant.

**Table 3:** Gustatory taste score according to the severity of COVID-19 infection.

	Disease Form		
Taste	Minor/Moderate (*n* = 30)	Severe (*n* = 59)	*p* Value
Total Score	7.96 ± 3.30	6.86 ± 3.13	ns
Bitter	2.36 ± 1.44	1.66 ± 1.28	0.021
Sour	1.36 ± 0.85	0.57 ± 0.81	<0.001
Salty	1.83 ± 1.44	2.01 ± 1.34	ns
Sweet	2.40 ± 1.42	2.61 ± 1.27	ns

The alteration of bitter and sweet tastes was significantly associated with an increase in IL-1β levels (*p* = 0.018 and *p* = 0.004, ns: no significant) (Table 4).

**Table 4:** Serum levels of inflammatory markers and taste disorders.

	[IL-1β] pg/mL	[IL-6] pg/mL	[TNF-α] pg/mL	CRP (mg/L)
Sweet Ageusia/hypo (*n* = 20) Normogeusia (*n* = 69) *p* value	10.75 ± 1.2 7.12 ± 2.32 0.004	19.18 ± 20.4 30.08 ± 63.85 ns	1.4 ± 0.25 1.35 ± 0.49 ns	60.04 ± 26.65 40.9 ± 7.17 ns
Salty Ageusia/hypo (*n* = 34) Normogeusia (*n* = 55) *p* value	8.54 ± 2.16 7.07 ± 2.35 ns	12.93 ± 10.81 28.60 ± 36.87 ns	1.35 ± 0.28 1.36 ± 0.48 ns	85.58 ± 66.92 22.50 ± 18.75 ns
Sour Ageusia/hypo (*n =* 63) Normogeusia (*n* = 26) *p* value	7.6 ± 1.5 7.59 ± 2.24 ns	25.04 ± 21.49 28.69 ± 47.43 ns	1.31 ± 0.28 1.38 ± 0.48 ns	84.16 ± 72.58 86.15 ± 78.35 ns
Bitter Ageusia/hypo (*n* = 20) Normogeusia (*n* = 69) *p* value	10.83 ± 13.44 6.83 ± 1.99 0.018	22.64 ± 22.5 29.10 ± 64.29 ns	1.43 ± 0.64 1.34 ± 0.39 ns	96.8 ± 82.29 80.51 ± 67.52 ns
Total score Ageusia/hypo *(n =* 55) Normogeusia (*n* = 34) *p* value	8.05 ± 8.39 7.22 ± 2.00 ns	27.16 ± 65.2 28.44 ± 43.18 ns	1.39 ± 0.49 1.33 ± 0.42 ns	88.36 ± 73.28 78.23 ± 71.54 ns

ns: no significant difference.

## Data Availability

Data can be made available by the corresponding author upon reasonable request.
